# Enhancing the electrical readout of the spin-dependent recombination current in SiC JFETs for EDMR based magnetometry using a tandem (de-)modulation technique

**DOI:** 10.1038/s41598-024-64595-3

**Published:** 2024-06-20

**Authors:** Andreas Gottscholl, Hannes Kraus, Thomas Aichinger, Corey J. Cochrane

**Affiliations:** 1 NASA Jet Propulsion Laboratory, California Institute of Technology, 4800 Oak Grove Dr, Pasadena, CA 91104 USA; 2 Infineon Technologies Austria AG, Siemensstraße 2, 9500 Villach, Austria

**Keywords:** Applied physics, Quantum metrology

## Abstract

Electrically detected magnetic resonance (EDMR) is a promising method to readout spins in miniaturized devices utilized as quantum magnetometers. However, the sensitivity has remained challenging. In this study, we present a tandem (de-)modulation technique based on a combination of magnetic field and radio frequency modulation. By enabling higher demodulation frequencies to avoid 1/f-noise, enhancing self-calibration capabilities, and eliminating background signals by 3 orders of magnitude, this technique represents a significant advancement in the field of EDMR-based sensors. This novel approach paves the way for EDMR being the ideal candidate for ultra-sensitive magnetometry at ambient conditions without any optical components, which brings it one step closer to a chip-based quantum sensor for future applications.

## Introduction

Magnetometers hold a versatile range of applications, from field mapping and GPS-denied navigation to medical imaging and nondestructive material analysis^1–3^. They also play a pivotal role in less apparent domains such as dark matter and dark energy detection^4^. In addition to their wide utility and sensitivity to various physical phenomena, magnetometers serve as indispensable instruments for space missions, aiding in the prediction of space weather and the study of the interior of planetary bodies^5,6^. Current magnetometers like fluxgates and optically pumped atomic gas magnetometers exhibit impressive sensitivity and reliability; however, fluxgates suffer from drift and are not absolute^7^, while atomic gas magnetometers are complex as they require optics that can be very sensitive to temperature variations.

In response to these limitations, ongoing research explores alternative magnetometer technologies. Superconducting Quantum Interference Devices (SQUIDs)^8^ offer enhanced sensitivity but are hindered by their dependence on cryogenic cooling systems, making them less suitable for most space missions due to the deteriorated SWaP (size, weight and power) of its required cooling infrastructure. For many applications sensitivities on the order of 100–1000 $\frac{\text {pT}}{\sqrt{\text {Hz}}}$ suffice, a range within the capabilities of spin defect-based magnetometers (e.g. using NV diamond)^9^. These magnetometers excel due to their simplicity and compatibility with ambient operating conditions^10,11^. Furthermore, they exhibit radiation hardness and can withstand high temperatures, corrosive environments, and elevated pressures due to the use of its robust solid state materials, in which the spin centers are hosted and are not able to permeate like the gas in vapor cells^12–14^.

Spin defects are commonly accessed through techniques like Electron Paramagnetic Resonance (EPR), Optically Detected Magnetic Resonance (ODMR), and Electrically Detected Magnetic Resonance (EDMR). While EPR serves as a fundamental measurement technique, mainly for material studies, ODMR and EDMR offer optical and electrical readout capabilities, respectively. While ODMR, particularly with NV diamond, achieves the highest sensitivities^15^, it is hampered by the need for lasers, detectors, and increased power consumption, complicating its application for miniaturized sensors.

A more straightforward approach is EDMR, relying on the spin-dependent recombination (SDR) process and operating exclusively in the electrical domain, without the need for indirect processes such as optical absorption and emission^16^. While EDMR is traditionally performed at low magnetic fields to explore spin-dependent phenomena, recent efforts have extended its application to high magnetic field regimes, aiming to uncover new insights into material properties and spin dynamics^17^. Though EDMR may not currently achieve the same level of sensitivity as ODMR when using NV diamond, two potential approaches arise to improve its sensitivity: linewidth reduction and signal-to-noise ratio (SNR) enhancement. Linewidth reduction can be achieved through sample engineering, notably using isotopically purified samples^18^. SNR can be improved through various methods, including above bandgap excitation (UV illumination), controlled defect creation via radiation, common mode rejection (CMR) and different biasing methods to utilize e.g. the bipolar amplification effect (BAE) for EDMR^19–25^.

In this paper, we introduce a novel tandem (de-)modulation technique, performing two modulations—magnetic field modulation (BM) and RF amplitude modulation (AM)—while demodulating solely at a single frequency (sum or difference of the modulation frequencies). In contrast to tandem demodulation in other fields^26^ this technique does not need a second step demodulation with extra filtering and can easily be implemented within a virtual lock-in. Thus, this approach can be readily incorporated into existing EDMR setups and has the potential to be simply combined with established SNR enhancement techniques. This new measurement technique offers three significant advantages compared to the conventional magnetic field modulation-based approach:

**1. Higher demodulation frequencies:** The demodulation frequency is no longer constrained by the modulation coils, potentially enabling the reduction of 1/f noise until measurements are limited solely by shot noise.

**2. Reduced self-calibration requirements:** The calibration process, traditionally reliant on bias fields in the $10~\text {mT}$ range, may be accomplished with smaller fields. This technique is blind to the inherent spin-dependent recombination magnetoresistive response that arises at zero-magnetic field due to energy-level mixing, mitigating calibration challenges that are associated with its presence.

**3. Separation of demodulation frequency from modulation frequency:** This separation eliminates the background interference waveform arising from the modulation coil, which induces an offset current in the sensor that manifests itself as a magnetometer offset, resulting in an enhancement of the signal-to-background ratio (SBR) by a factor of 1000.

**Figure 1:**
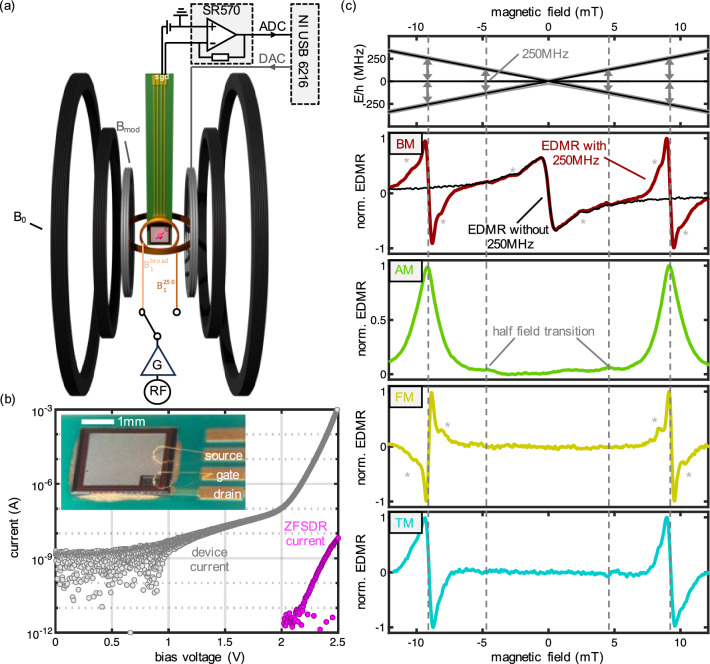
EDMR overview: (**a**) setup configuration of the different magnetic field coils. The offset coils (black) are in line with the magnetic field modulation coils (grey). The two RF coils for broadband (orange) and $250~\text {MHz}$ specifically (dark orange) are perpendicular. (**b**) JFET IV characteristics and sample photo (with 1mm scale bar). The JFET is wire bonded to apply a forward bias, which leads to the standard characteristics (grey). Between 2.1 and 2.5 V a zero-field spin-dependent current (ZFSDR) displayed in pink is observed. (**c**) EDMR measurements using different modulations: magnetic field modulation (BM), RF amplitude modulation (AM), frequency modulation (FM) and the novel tandem modulation (TM). The top subfigure shows the calculated energy level (units in MHz) of the involved triplet ($^{28}$Si in black, $^{29}$Si isotope in grey). The other subfigures present the various modulation techniques BM (red), AM (green), FM (yellow) and TM (cyan). Observable hyperfine peaks are marked with grey stars. All four measurements show the $250~\text {MHz}$ resonance peak and even the spin-forbidden half-field transition (light grey) is visible for BM, AM and TM.

## Basic concept

The novel tandem demodulation technique can be seamlessly integrated into a standard electrically detected magnetic resonance (EDMR) setup. Therefore, we will now focus on the main components of the setup as illustrated in Fig. 1a:

**Magnetic field components:** The magnetic field generation is achieved through a set of three Helmholtz coils, with two pairs serving for field offset control and one pair dedicated to modulation purposes.

**Radio frequency (RF) components:** The RF system comprises an RF source and an amplifier, essential for achieving the requisite $B_1$ fields. In this study, two coils were utilized, both oriented perpendicular to the offset magnetic field. Depending on the nature of the measurement, either a $250~\text {MHz}$ coil was employed to enhance the signal-to-noise ratio (SNR), owing to its strong $B_1$ field conversion, or a broadband antenna loop was utilized, offering a frequency range between $2~\text {MHz}$ and $250~\text {MHz}$ ($2~\text {MHz}$ is the lower limit of the RF amplifier employed).

**Silicon carbide (SiC) device for spin-dependent recombination:** The heart of the system is a wire-bonded SiC Junction Field-Effect Transistor (JFET) device (see “Methods” section and Aichinger et al.^27^ for sample details) with a positively biased gate. Due to the highly doped n-channel region, source and drain are shorted, thus, any port of the two can be used for current measurement, and the other one can be left floating.

In Fig. 1b, the IV characteristic of the JFET device is presented in grey, which is essentially the DC offset current during the EDMR measurements. For bias voltages ranging between 2.1 and $2.5\,\,\text {V}$, a magnetic field dependent current (pink symbols) due to spin-dependent recombination (SDR) of the involved electrons emerges when the device is close to zero magnetic field (see^28^). The effective total SDR current is defined as the integrated EDMR current divided by the full magnetic field range (see^16^ for details). In order to reveal these small magnetic field dependent current contributions, we added an transimpedance amplifier (Stanford Research System SR570) to the biasing scheme and recorded the output with an ADC (National Instruments USB 6216). To enhance the SNR, we modulate the magnetic field strength (grey magnetic field coils) with one of the DAC outputs. Using a virtual lock-in amplifier enables an extraction of current contributions oscillating with the modulation frequency, thus revealing magnetic field dependent contributions of the current. The best SNR can be obtained when applying $2.3\text {V}$, which is a trade-off between a high resonant SDR current and sufficiently low DC device current, the latter driving measurement noise.

We now want to focus on the different types of EDMR spectra: In the top of Fig. 1c we plotted in black the energy levels of the involved triplet and in grey the energy levels when a $^{29}$Si isotope is involved in the process (simulations conducted with EasySpin^29^ assuming a triplet $S=1$ with $g\approx 2$, $D=0$). As soon as a magnetic field is applied, the energy levels of the triplet is detuned. The EDMR signal versus the applied magnetic field is shown in Fig. 1c (black). Note that, due to magnetic field modulation, the observed EDMR spectrum is recorded as the first derivative of the EDMR response.

By introducing RF radiation at a specific frequency (e.g., $\nu =250~\text {MHz}$), additional energy transitions are induced. The associated energy transitions resulting from spin flips are illustrated in Fig. 1c (BM EDMR, red curve). The central main peak, as well as the two RF induced peaks (referred to here as resonant SDR peaks) have minute shoulders (marked with grey stars), attributed to hyperfine peaks of $^{29}Si$. A comprehensive analysis of BM EDMR spectra, and more detailed energy schemata can be found in Cochrane et al. and Harmon et al.^16,17,28^.

The resonant SDR transition can be leveraged to self-calibrate an EDMR-magnetometer, however, to characterize the transitions in the presence of the zero-field spin-dependent recombination (ZFSDR), requires a significant field bias to resolve the resonant SDR from the ZFSDR transitions. From an application perspective, large field biasing for near-zero DC magnetometry would significantly drive instrument size, weight and power (SWaP), parameters of utmost importance, on par with sensitivity, for space and defense applications. One goal of this work is to reduce this bias requirement for self-calibrated magnetometer operation.

To provide an overview of all involved modulation techniques of this study, we proceed with amplitude modulation (AM) of the RF, revealing contributions exclusively associated with RF interactions. Due to the square wave modulation of the RF amplitude and continuous magnetic field sweeping, we observe the absorption line-shape rather than the first-derivative line-shape characteristic of $B_0$ modulation (center measurement shown in green in Fig. 1c).

We show below that the first derivative zero-crossing of the EDMR signal amplitude is indispensable to implement resonance locking^30^, and thus AM zero order responses are not conducive for easy magnetometer implementation. One alternative is frequency modulation (FM), as depicted in Fig. 1c (FM, yellow curve)^31^. Like AM EDMR, FM EDMR avoids a signal contribution from ZFSDR at zero field, however now offering a derivative signal. The phase shift of $\pi$ between the two resonant SDR peaks occurs due to the symmetric energy level splitting around $B_0=0$, resulting in a mirrored spectrum when only using the frequency modulation technique. While this mode can be used for a scalar measurement of magnetic fields, the signal does not contain any information on the magnetic field orientation.

This is due to detuning of the RF energy from resonance, unlike modulating magnetic field, does not shift the Zeeman energy levels ($E=g \mu _{\textrm{Bohr}} {\textbf{B}}$) themselves. However, to determine external field orientation, we have to probe energy level shift in three directions, which is unfeasible with diffuse RF field. To recover field directionality, magnetic field modulation has to be implemented as demonstrated in^16^.

Therefore, we have developed the tandem modulation (TM) technique (displayed in cyan in Fig. 1c). TM EDMR combines the advantages of magnetic field modulation (BM) and RF modulation (AM or FM). TM corresponds to the product of BM and AM spectra but is achieved through straightforward demodulation at a single frequency. This results in a spectrum similar to the BM EDMR spectra, but disabling the ZFSDR signal (as can be seen by comparing the cyan and red spectra in subfigure c). This phenomenon arises because TM involves magnetic fields and RF simultaneously, making it sensitive only to transitions that require both.

While in ODMR using NV centers, a bias can result in many transitions being addressed simultaneously (see Schloss et al.^9^), in EDMR, we cannot utilize both the resonant SDR transition and the ZFSDR transition at the same time due to the requirement of zero vs. nonzero magnetic field. Therefore, it is advantageous to bring the transitions as close as possible without causing them to overlap. While BM EDMR without RF enables only the ZFSDR transition (shown in black), TM EDMR now offers a new possibility to address exclusively the resonant SDR transitions. Consequently, we can selectively address either ZFSDR or resonant SDR for measurement and calibration purposes, which will be further discussed in the “Tandem modulation and demodulation” section of the manuscript.

Note that each of the subfigures in Fig. 1c were recorded within a 90-second interval to compare the quality of the data of each modulation method. Any additional noise observed in the TM spectrum can be attributed to phase noise, stemming from the lack of synchronization between the two modulation frequencies. The observed current noise $\delta I$ in the signal *I* is directly projected onto the magnetic field axis *B* using the observed slope ($\frac{\Delta I}{\Delta B}$) of the resonant SDR signal to obtain the sensitivity $\frac{\delta B}{\sqrt{\Delta f}}=\frac{\Delta B}{\Delta I}\delta I \sqrt{\Delta t}$. Here, $\Delta f$ is the standard bandwidth of $1~\text {Hz}$ given by the chosen acquisition time $\Delta t=1~\text {s}$. We estimate the sensitivity of the different modulation techniques to be $0.6\frac{\mu \text {T}}{\sqrt{\text {Hz}}}$ (BM), $1.3\frac{\mu \text {T}}{\sqrt{\text {Hz}}}$ (FM), and $1.8\frac{\mu \text {T}}{\sqrt{\text {Hz}}}$ (TM). Please note that these are only proof-of-concept results to provide an overview of all used modulation techniques in this paper. While BM performs twice as good as FM and three times as good as TM, we want to emphasize that TM will perform as good as FM when using two sideband frequencies (as mentioned later in the “Tandem modulation and demodulation” section). Furthermore, the true benefits of TM will be realized when higher demodulation frequencies are employed and devices with stronger signals are explored, as TM will then enable access to the full dynamical range, as explained in the “Enhancement of the signal background ratio” section. For an additional discussion about the observed linewidth see the “Method” section.

## EDMR with arbitrary RF

**Figure 2:**
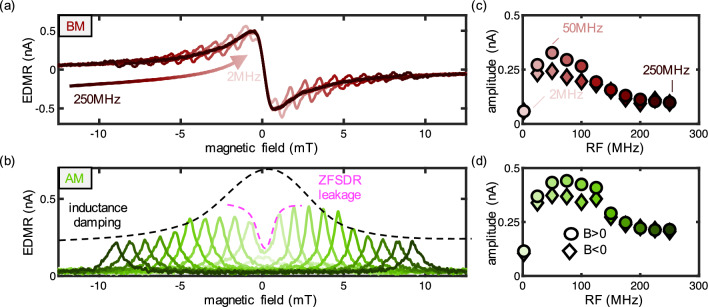
Low frequency EDMR with broadband antenna loop: (**a**) BM EDMR with RF between $\nu$ = 2 MHz and $\nu$ = 250 MHz. The signal is dominated by the standard ZFSDR signal. (**b**) AM EDMR with different frequencies between $\nu$ = 2 MHz (light green) and $\nu$ = 250 MHz (dark green)to reveal only the RF involved transitions. The peaks are damped by the ZFSDR leakage (pink) and the high impedance of the loop antenna due to its high inductance (black). (**c**) and (**d**) peak amplitude of BM and AM, respectively. The broadband antenna is most efficient at $\nu =50~\text {MHz}$ which is used for low frequency proof of concept measurements.

The measurements presented in Fig. 1c were conducted with an RF frequency of $\nu$=250MHz, corresponding to a bias field of 9mT. However, this level of bias field may not be practical for miniaturized devices. To enable the use of smaller fields, we must employ lower RF frequencies. The key advantage of lower RF frequencies lies in the reduced separation between the two resonant SDR peaks, thereby lowering the required field strength for offset coils. In Fig. 2a, we showcase EDMR spectra acquired using the broadband loop antenna, spanning RF frequencies from 2 to 250MHz.

Notably, due to the less intense $B_1$ field generated by this antenna, the resonant EDMR peaks appear smaller in amplitude compared to the central ZFSDR peak. A critical challenge emerges when using resonant EDMR transitions at lower fields: the EDMR spectrum becomes primarily dominated by the ZFSDR transition, resulting in an impractical utilization of the resonant SDR peaks. As previously discussed, we can introduce RF modulation to selectively highlight the resonant SDR contributions. However, the application of AM (see Fig. 2b) yields a non-derivative behavior, making it unsuitable for simple resonance locking. Deviations from the resonance mode would result in the same sign of the EDMR current, thereby preventing the differentiation of the necessary field strength. Additionally, AM alone lacks the ability to function as a vector magnetometer, as the demodulated signal does not contain information about the field orientation.

Despite these disadvantages of AM, it enables an analysis of the broadband antenna’s capabilities, demonstrating the feasibility of using lower frequencies despite the strong ZFSDR signal. ZFSDR only damps the signal around zero field, since this effect introduces leakage channels that reduce the EDMR current when employing the lock-in technique. This effect is also known in hole-burning spectroscopy, which has its roots in laser spectroscopy, and it has been observed for various other materials using optically detected magnetic resonance (ODMR) and electroluminescence detected magnetic resonance (ELDMR) with two frequencies^32–34^.

The peak amplitudes are analyzed in Fig. 2c, d, pertaining to the BM and AM methods, respectively. As mentioned the reduction in signal amplitude at lower frequencies can be attributed to the ZFSDR signal. In contrast, the damping observed at higher frequencies results from the increased impedance of the coil due to its inductance: $B_1\propto \frac{1}{Z}=\frac{1}{\sqrt{R_{DC}^2+X_L^2}},X_L\propto f_B$. At higher frequencies, the coil becomes less efficient, leading to a reduced $B_1$ field for the same RF power. Consequently, we employ frequencies around $\nu =50~\text {MHz}$ for low magnetic field bias measurements, as they yield maximum performance. For higher frequencies, we continue to utilize the $\nu =250~\text {MHz}$ coil due to its superior performance.

Remarkably, we observe a discrepancy in peak amplitudes between positive and negative resonant SDR peaks for both BM and AM methods. The peaks for positive magnetic field values appear larger than their negative counterparts across all measurements within this study. We can exclude hysteresis effect since an identical behavior for the positive magnetic field value is observed when the magnetic field is swept from the opposite direction. The origin of this phenomenon remains unclear and demands further examination in future studies using different setups.

## Tandem modulation and demodulation

Following the efficiency assessment of the broadband antenna, this section focuses on the evaluation of the proposed TM technique at both high ($\nu =250~\text {MHz}$) and low ($\nu =50~\text {MHz}$) RF frequencies. The underlying principle of this technique centers on leveraging the benefits of both BM and AM, similar to fundamental modulation techniques used in communication. In this approach, one modulation frequency serves as the carrier frequency, while the other modulates the carrier signal. The resulting signal manifests itself in two sidebands. For this study, we used $f_B=500~\text {Hz}$ as the magnetic field modulation frequency and $f_{RF}=5100~\text {Hz}$ as the amplitude modulation frequency of the RF signal. The choice of AM modulation frequency is set an order of magnitude higher than BM to clearly differentiate them. Furthermore, a small offset of $100~\text {Hz}$ was chosen to avoid higher harmonic interference (e.g., 10x $500~\text {Hz}$). The standard demodulation frequency employed is therefore the sum of both modulation frequencies, totaling $f_{demod}^+=5600~\text {Hz}$. Importantly, we anticipate exploring various modulation and demodulation frequencies in the following section to demonstrate the technique’s adaptability across different combinations.

**Figure 3:**
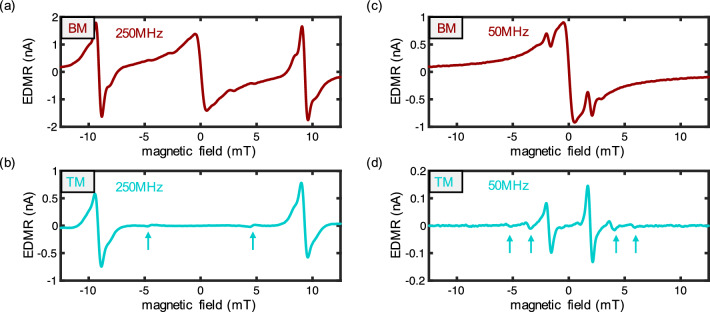
BM vs TM EDMR: (**a**) standard BM (red) EDMR for $\nu =250~\text {MHz}$. (**b**) novel TM (cyan) EDMR which avoids the strong ZFSDR signal peak. Interestingly, the half-field transitions (cyan arrows) show a phase flip of pi compared to the main resonant SDR peak transitions indicating different populations of the $m_s=+1$ and $m_s=-1$ state. (**c**) BM EDMR with $\nu =50~\text {MHz}$. (**d**) TM EDMR with $50~\text {MHz}$. The novel TM EDMR approach is also applicable to small fields (/frequencies) when the signal is usually dominated by ZFSDR. Multiple hyperfine peaks can be observed (cyan arrows) which are less suppressed than the resonant SDR peak located within the ZFSDR signal.

In Fig. 3a, b, we present the results for the BM (red) and TM (cyan) techniques, respectively. Figure 3b notably showcases only the resonant SDR peaks with the characteristic first derivative behavior typical of standard BM. Notably, even the half-field transitions around 5mT are discernible (indicated by the blue arrows) and show a phase difference of pi. This might provide access to the population difference of the triple $m_s=-1$ and $m_s=+1$ state, but is beyond the scope of this work. Comparing TM with BM clearly shows an overall signal amplitude reduction for the TM measurements due to the lower effective RF power (resulting from square wave modulation instead of constant RF power) and the signal’s distribution between the two sidebands. Remarkably, an additional second sideband could be employed concurrently, theoretically leading to an SNR enhancement of $\sqrt{2}$ (see^35^for comparable approach) since the random noise of *N* measurements can be averaged and therefore reduced by a factor of $\sqrt{N}$. Notably, taking this factor into account TM would result in the same sensitivity as previously observed for FM: $\frac{\delta B_{TM}}{\sqrt{\Delta f}}/\sqrt{2}\approx 1.3\frac{\mu \text {T}}{\sqrt{\text {Hz}}}=\frac{\delta B_{FM}}{\sqrt{\Delta f}}$. It is important to note that the two modulation frequencies of the here presented TM measurements were not synchronized, introducing some additional phase noise. Consequently, the in-phase and out-of-phase data were phase corrected after demodulation.

The primary motivation behind introducing the tandem modulation and demodulation technique was to mitigate the substantial signal arising from the ZFSDR effect. In Fig. 3c and d, we conducted analogous measurements with lower frequencies, where the ZFSDR response dominates. Employing TM, we observe two distinct resonances without the interference of the substantial ZFSDR signal. The ZFSDR primarily affects the signal’s amplitude, serving as a leakage channel for the spins. Despite the minor reduction in signal amplitude due to this effect, resonance transitions are now available for potential use in future magnetometer applications, particularly for coil calibration, as elaborated in Cochrane et al.^16^.

## Sideband configurations for TM

**Figure 4:**
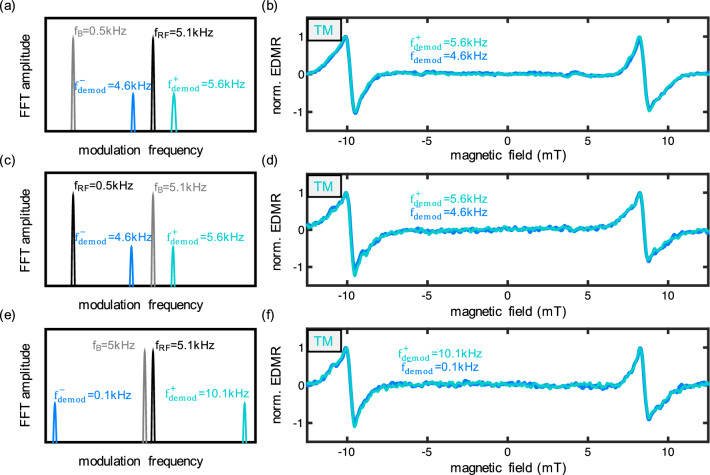
TM EDMR with different (de-)modulation frequencies: (**a**) FFT illustration of the standard TM EDMR of this paper. (**b**) TM EDMR spectra with higher (cyan) and lower (blue) demodulation frequency. The two spectra are plotted over each other to highlight the similarities of both sidebands. (**c**) and (**d**) TM EDMR with high BM and low AM. (**e**) and (**f**) with high BM and high AM leading to a low sideband (blue) and a very high sideband (cyan). Especially this approach is attractive since it can bring the demodulation frequency far above the operation regime of magnetic field coils towards the shot-noise limit.

**Figure 5:**
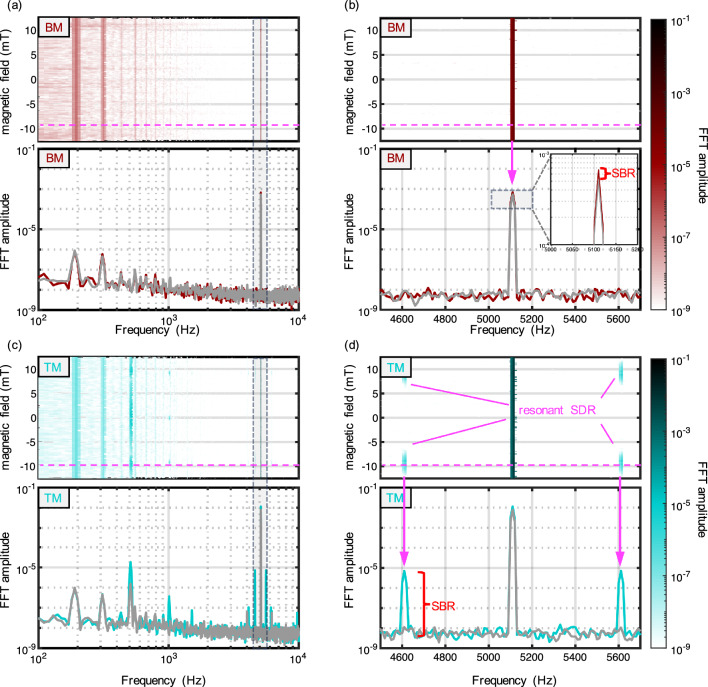
FFTs during EDMR (before demodulation): (**a**) FFT of BM EDMR (red). The colormap represents the absolute value of the FFT during a BM with $f_B$ = 5.1 kHz with a cross sections in blue (on resonance) and grey (off resonance = noise floor). (**b**) Zoom of BM EDMR revealing a large background signal at the demodulation frequency. (**c**) FFT of TM EDMR (cyan). TM with $f_B$ = 500 Hz and $f_{RF}=5.1~\text {kHz}$ leading to sideband in resonance. (**d**) Zoom of TM EDMR revealing the resonance peak far above the background noise.

After demonstrating the TM technique for arbitrary RF frequencies, we now focus on examining the possibilities offered by arbitrary modulation frequencies. As previously noted, TM generates two sidebands at frequencies $|f_{RF}\pm f_B|$. In all measurements in this paper, we utilized $f_B$ at $500~\text {Hz}$ and $f_{RF}$ at $5.1~\text {kHz}$ unless otherwise stated. The frequency configuration is visually depicted in Fig. 4a. It is important to mention that the measured fast Fourier transform (FFT) is more intricate, encompassing higher harmonics, interference frequencies from the line voltage, and the demodulation peaks are too narrow compared to the entire frequency spectrum. A comprehensive FFT is later presented in Fig. 5, but for the current analysis, a simplified illustration suffices.

In Fig. 4b, we provide a comparison of both sidebands, plotted over each other to highlight the similarity of the result. At both $f_{demod}^+=5.6~\text {kHz}$ and $f_{demod}^-=4.6~\text {kHz}$, clear EDMR signals emerge from the two resonant SDR transitions. To underscore the versatility of the TM technique, we interchanged the frequencies of $f_{RF}$ and $f_B$ in Fig. 4c and d. The signal exhibits increased noise, attributed to the modulation coil’s lack of optimization for frequencies above 1kHz, which leads to a reduced effective modulation depth.

The true advantage of employing arbitrary modulation and demodulation frequencies becomes apparent when both frequencies are positioned to higher frequencies as showcased in Fig. 4e and f for the 5kHz regime. For proof of concept, we can demodulate the signal at $f_{demod}^-=100~\text {Hz}$, although this has no practical benefit due to the higher noise floor. The real benefit emerges in the higher sideband ($f_{demod}^+=10.1~\text {kHz}$): with TM we are able to demodulate the signal within a range where the magnetic field coil becomes highly inefficient. Thus, TM empowers devices to operate far beyond the conventional modulation coil’s operational range. In principle, this approach can entirely circumvent 1/f noise, making the measurement predominantly limited by shot noise. The maximum demodulation frequency is constrained by $f_B^{max} + f_{RF}^{max}$, which is limited by the inductance of the coil (or the RLC circuit) and the bandwidth limitations of the RF antenna.

## Enhancement of the signal background ratio

The TM technique not only shifts the signal to a less noisy frequency regime but also effectively separates it (in frequency) from the strong modulation-driven background current induced in the sensor arising from electromagnetic pickup of the electrical circuit. In Fig. 5a, we present the FFT of a typical BM EDMR measurement before demodulation. The upper figure exhibits a color map representing the FFT during a magnetic field sweep, while the lower Fig. 5a displays a cross-section at the on-resonance (red) and off-resonance (grey). Conventional BM measurements typically exhibit the characteristic 1/f noise reduction at higher frequencies. However, despite the high SNR, the Signal-to-Background Ratio (SBR) remains significantly smaller than the SNR value. In Fig. 5b, we provide a zoomed-in view of the modulation frequency, where the SNR compared to the noise floor reaches approximately $10^5$, but the SBR right at the demodulation frequency remains less than 2. This background, frequently observed in lock-in measurements, becomes especially critical when attempting to use zero crossings for sensor applications. The unwanted background not only compromises sensor accuracy but also limits sensitivity, as the digitizer’s dynamic range is often consumed by this background. Without the background, the dynamic range can be fine-tuned to the noise floor, enabling the detection of smaller external fields. Shielding can help mitigate this background, since it is primarily generated by induced currents from the modulation coils into the sensor probe wires.

With the TM technique, however, we can decouple the demodulation from the modulation frequencies, as illustrated in Fig. 5c. The modulation frequencies create a distinct, strong peak in the cross-section. This time, however, the resonant SDR peaks emerge from the noise floor when measured via the sidebands. A closer examination is provided in Fig. 5d. Even in the color map, we observe EDMR resonances as strong peaks. The cross-section of the zoomed-in region reveals a huge SBR of approximately 2000, which is approximately three orders of magnitude stronger than the peak seen in Fig. 5b. It is important to note that the amplitudes of these peaks are still 2 orders smaller than those created by BM and 3 orders of magnitude smaller than the $f_{RF}$ peak. Nevertheless, no discernible background is observed above the noise floor with this technique.

Incorporating a filter that blocks the modulation peak at $f_{RF}=5.1~\text {kHz}$ would allow for dynamic range adjustment solely to the resonance signal, significantly enhancing sensitivity by up to 3 orders of magnitude, assuming the current sensitivity is limited by the large background signal. This capability offers opportunities for enhanced signal detection in applications where background interference has traditionally posed a challenge. As a side note, we want to mention that TM can be realized not only by using the direct sideband frequencies (as depicted in subfigure d) but also through their higher harmonics. In subfigure c, multiple higher harmonics are visible, including those of the sidebands as well as those of the magnetic field modulation (see $1~\text {kHz}$). It’s important to note that spectra obtained from these demodulation frequencies offer additional benefits, though they are not as straightforward to use as detailed in Cochrane et al.^16^.

## Zero field application and vector magnetometer mode

**Figure 6:**
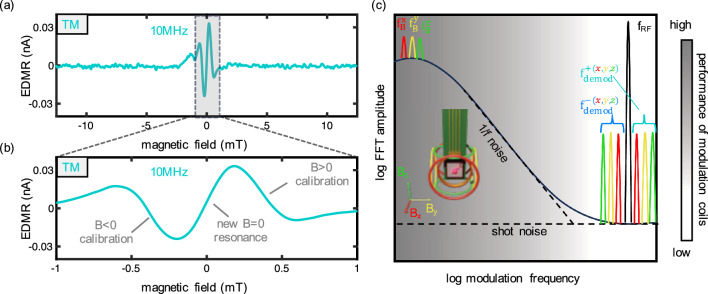
TM EDMR for vector magnetometry. (**a**) and (**b**) Low TM EDMR with $\nu =10~\text {MHz}$. An overlap of the two resonant SDR peaks leads to a new $B=0$ resonance condition which can be employed for magnetometry. (**c**) Illustration of an extension to three dimensions. $f_B^x$, $f_B^y$ and $f_B^z$ have distinguishable values but their maximum frequency is limited by the performance of the modulation coils. Using a common $f_{RF}$ leads to high frequency resonant transitions in the shot noise regime and distinguishable for orientation information.

The tandem modulation (TM) technique presented here primarily focuses on the resonant SDR transitions, which are crucial for the remote self-calibration of the solid-state magnetometer under development. In our final exploration, we examine whether this technique, designed for background avoidance, can also be applied at zero magnetic field, thus avoiding the zero-field mixing signal completely. Instead of relying on the ZFSDR to sense near-zero magnetic fields, we deliberately overlap the two resonant transitions, resulting in a new “resonant” slope centered on zero magnetic field. To achieve this, we select a low RF frequency ($\nu =10~\text {MHz}$), as depicted in Fig. 6a. A closer look at the two overlapped first derivative peaks is presented in Fig. 6b. Due to the overlap we create a new magnetic field dependent zero-crossing manifesting as a linear slope at zero field, with absolutely no offset in the measurement. When the spin system resides between both resonant transitions, the EDMR response remains at zero. Any detuning resulting from an external field induces a positive or negative TM EDMR current, contingent upon the field’s direction. Notably, the required field for calibration is now reduce by a factor of 25, thus, enabling self-calibrating EDMR devices with much smaller coils. This concept can potentially be extended to a vector mode by employing multiple pairs of modulation coils, one pair for each orientation (see illustration in Fig. 6c based on Cochrane et al. ^16^). Each orientation can be modulated with a distinct $f_B$, as previously described. A common RF frequency of $\nu =10~\text {MHz}$ can be used for all orientations simultaneously. By employing a high $f_{RF}$, exceeding the 1/f noise regime, all $f_B$ frequencies are shifted into this regime, with sidebands symmetrically mirrored around $f_{RF}$. Demodulating each of these frequencies provides access to all three orientations of an external field.

As long as the external field remains within the linear regime (i.e., below the modulation depths), the EDMR current of each demodulation is directly proportional to the field’s strength in that particular orientation. For external fields that exceed this linear range, compensation fields can be applied, as elaborated in Cochrane et al.^16^. The originally proposed self-calibration process can be executed by introducing an additional current to the coil to align it with one of the resonant SDR peaks (e.g. $0.36~\text {mT}$ for $\nu =10~\text {MHz}$).

## Conclusion

In this work, we introduced a novel tandem demodulation technique for EDMR that effectively addresses critical challenges in enhancing sensitivity for miniaturized quantum devices, particularly magnetometers. Our innovative approach enables the use of higher demodulation frequencies, effectively mitigating the influence of 1/f noise. Furthermore, it allows for arbitrary bias fields during calibration, even within the main peak of the EDMR spectrum. Most importantly, this technique eliminates background interference, enabling the full utilization of the sensor’s dynamical range. This does not only promises advancements in EDMR but also in related measurement techniques such as EPR, ODMR, and ELDMR. In addition to applying tandem (de-)modulation to other measurement techniques, we can further benefit by using different EDMR samples, such as SiC MOSFETs, where BAE biasing schemes can significantly improve signal strength^21^. This enhancement in combination with the here presented tandem (de-)modulation enables the full utilization of the dynamic range. When combined with complementary signal-enhancing methodologies^19,20,22^ such as UV irradiation and CMR, as well as sample engineering^36,37^ involving isotopically purified samples, EDMR emerges as an essential technology for miniaturized quantum devices^38^.

## Methods

SiC Sample: All measurements were performed with a 4H SiC Junction Field Effect Transistor (JFET) device. The n-type region has been doped with nitrogen with a dopant level of $5\cdot 10^{17}~\text {cm}^{-3}$ and the p-type region has been doped with a high concentration of aluminum (see^27^ for more details).

IV Measurement: To characterize the SiC JFET, we wire-bonded it onto a self-designed PCB. The IV measurements were performed using a Keysight B2901B, with which we biased the gate and grounded the drain.

EDMR Measurement: The EDMR measurements were conducted using a homebuilt EDMR spectrometer. The sample was biased with 2.3V, utilizing the internal bias of the transimpedance amplifier (Stanford Research Systems Model SR570). We used the 6dB bandwidth mode with cut-off frequencies outside of the demodulation range to reduce temperature drifts at low frequencies and potential high frequency noise without attenuating the EDMR signal itself. Additionally, we used the high bandwidth mode of the amplifier with moderate sensitivities to ensure no artificial distortion of the signal. The voltage output was fed into a National Instruments USB 6216 DAQ, where we used a software lock-in to demodulate the EDMR signal. The NI USB DAQ controlled the homebuilt spectrometer to sweep the current in the 3D-printed Helmholtz coils (diameters $18.5~\text {cm}$ and $13~\text {cm}$), thereby sweeping the magnetic field between $\pm 12.5~\text {mT}$. A Hall sensor monitored the current magnetic field value, which was fed into the magnetic field controller via a PI loop. To induce resonant transitions at non-zero fields, we applied radio frequencies (Stanford Research Systems Model SG382) via RF coils (Doty Scientific coil with a coil matching network and custom-made coil). To achieve a sufficiently high $B_1$ field, we amplified the RF signal using a Mini-Circuits ZHL-1A amplifier. All modulation techniques that utilize magnetic field modulation (both BM and TM) were carried out using a sinusoidal modulation with an amplitude of $0.5~\text {mT}$. This amplitude represents a compromise between achieving a high signal amplitude and only slightly overmodulating the linewidth. The AM measurements were conducted using a square wave modulation of the RF amplitude to toggle the resonance condition on and off. For FM, both tuning and detuning of the resonance condition are necessary; therefore, a standard sinusoidal modulation is employed. Notably, we used a modulation depth of 2MHz, which is the maximum of the SG382 RF source within the $250~\text {MHz}$ range. This corresponds to a magnetic field amplitude of $0.07~\text {mT}$, nearly an order of magnitude smaller than the amplitude used in BM. Consequently, the linewidth of the FM measurement (see Fig. 1c) is constrained only by the natural linewidth, rendering it narrower than the linewidths observed in BM and TM measurements.

## Data Availability

The data that support the findings of this study are available from the corresponding author upon reasonable request.
